# Efficacy and Safety of an Everolimus- vs. a Mycophenolate Mofetil-Based Regimen in Pediatric Renal Transplant Recipients

**DOI:** 10.1371/journal.pone.0135439

**Published:** 2015-09-25

**Authors:** Lena Caroline Brunkhorst, Alexander Fichtner, Britta Höcker, Greta Burmeister, Thurid Ahlenstiel-Grunow, Kai Krupka, Martin Bald, Antonia Zapf, Burkhard Tönshoff, Lars Pape

**Affiliations:** 1 Department of Pediatric Nephrology, Hannover Medical School, Hannover, Germany; 2 Department of Pediatrics I, University Children's Hospital Heidelberg, Heidelberg, Germany; 3 Department of Pediatric Nephrology, Olgahospital, Stuttgart, Germany; 4 Department of Visceral and Transplant Surgery, University Hospital of Schleswig-Holstein, Kiel, Germany; 5 Department of Biostatistics, University Clinic of Göttingen, Göttingen, Germany; University of Toledo, UNITED STATES

## Abstract

**Introduction:**

Data on the efficacy and safety of everolimus in pediatric renal transplantation compared to other immunosuppressive regimens are scarce.

**Patients/Methods:**

We therefore performed a multicenter, observational, matched cohort study over 4 years post-transplant in 35 patients on everolimus plus low-dose cyclosporine, who were matched (1:2) with a control group of 70 children receiving a standard-dose calcineurin-inhibitor- and mycophenolate mofetil-based regimen.

**Results:**

Corticosteroids were withdrawn in 83% in the everolimus vs. 39% in the control group (p<0.001). Patient and graft survival were comparable. The rate of biopsy-proven acute rejection episodes Banff score ≥ IA during the first year post-transplant was 6% in the everolimus vs. 13% in the control group (p = 0.23). The rate of de novo donor-specific HLA antibodies (11% in everolimus, 18% in controls) was comparable (p = 0.55). At 4 years post-transplant, mean eGFR in the everolimus group was 56±33 ml/min per 1.73 m² vs. 63±22 ml/min per 1.73 m² in the control group (p = 0.14). Everolimus therapy was associated with less BK polyomavirus replication (3% vs. 17% in controls; p = 0.04), but with a higher percentage of arterial hypertension and more hyperlipidemia (p<0.001).

**Conclusion:**

In pediatric renal transplantation, an everolimus-based regimen with low-dose cyclosporine yields comparable four year results as a standard regimen, but with a different side effect profile.

## Introduction

The short-term outcome following renal transplantation in pediatric patients with the current immunosuppressive regimens is excellent, but long-term graft survival has not improved to the same extent. The nephrotoxicity of calcineurin inhibitors (CNIs) may contribute to chronic allograft dysfunction. It has previously been shown in adult renal transplantation that the introduction of everolimus (EVR), a mammalian target of rapamycin (mTOR) inhibitor, may facilitate CNI minimization while maintaining sufficient immunosuppressive efficacy [1]. EVR targets the mTOR complex 1 in the signaling pathway of T cell growth factors, thereby hindering the proliferation of antigen-activated T cells. In the pediatric transplant patient population, only single-arm trials on EVR in conjunction with reduced dose cyclosporine microemulsion (CsA) have been published [2–5]. This regimen has potential advantages such as less CNI-induced side effects, particularly less chronic nephrotoxicity, and less steroid-related side effects, but may be associated with EVR-associated side effects such as anemia, dyslipidemia and impaired wound healing [6–8]. Because of the single-arm design of our previously published study, this was difficult to interpret. We therefore performed now a multicenter, retrospective cohort study in 105 pediatric renal transplant recipients on the efficacy and safety of an EVR-based regimen over 4 years post-transplant compared to a matched control group receiving a standard-dose CNI- and mycophenolate mofetil (MMF)-based regimen.

## Patients and Methods

### Study design

This was a multicenter, retrospective, matched cohort study in 105 pediatric renal transplant recipients. The EVR group (n = 35) was treated at Hannover Medical School. To avoid a selection bias, all children and adolescents with a 4 year follow-up who received a first or second AB0-compatible renal allograft with EVR-based immunosuppressive therapy between 11/2006-12/2009 were included (35 of a total of 41 transplantations performed). Within this time frame only six patients with a similar risk profile were initially treated with an MMF-based immunosuppressive regimen (combined with tacrolimus (TAC) or CsA) in Hannover, because they were referred for transplantation to Hannover from other centers with no experience with EVR, and long-term follow-up was performed locally. There was no selection for EVR treatment according to the immunological risk prior to transplantation. The EVR patients were retrospectively matched with two controls each, from two centers (University Children’s Hospital Heidelberg (n = 57); Children’s Hospital Stuttgart (n = 13)), who had received a first or second renal allograft in the same time period as the EVR group (2004–10). For each patient in the EVR group, two case-control counterparts with a minimum observation time of 4 years were identified using the CERTAIN Registry by means of the following five matching criteria: (i) age at transplantation, (ii) graft source (living donation or donation from a deceased donor), (iii) first or second transplant, (iv) gender, (v) pre-emptive transplantation or prior dialysis. Patients with potentially recurring primary renal diseases (focal segmental glomerulosclerosis (FSGS)) (n = 2) or membranoproliferative glomerulonephritis (n = 1) were included in the EVR group as well as in the control group (FSGS, n = 1). There was no lower age limit.

Of the 35 EVR-treated children, 33 (94%) received induction therapy with basiliximab on days 0 and 4. Basiliximab treatment was avoided in 2 children who had received basiliximab previously; these patients did not receive any other induction therapy. Patients received 300 mg prednisolone/m^2^ body surface area (BSA) intravenously at the time of engrafting. The mean prednisolone doses at month 1 and 6 post-transplant were 25±7.8 and 6.0±2.5 mg/m^2^ BSA per day, respectively. Prednisolone was discontinued in 29/35 (83%) patients at 10.3±1.9 months post-transplant. Prednisolone treatment was only maintained in patients with a history of acute rejection. CsA microemusion was administered. As EVR therapy was guided by trough level monitoring, no C2 monitoring for CsA was used. CsA target trough levels were 50–100 μg/L in the first 6 months post-transplant and 25–75 μg/L thereafter. EVR target trough levels were 3–6 ng/mL over the entire study period. The CsA and EVR doses and corresponding trough levels, measured by mass spectrometry, are given in Table 1. MMF was not part of the immunosuppressive regimen of the EVR group.

**Table 1:** Immunosuppressive therapy (mean doses per day and corresponding trough levels).

**EVR group**		
**Month 1**	**Month 12**	**Month 48**
CsA dose [mg/m^2^]	CsA dose [mg/m^2^]	CsA dose [mg/m^2^]
270 ± 56	103 ± 26	102 ± 16
CsA-C_0_ [μg/L]	CsA-C_0_ [μg/L]	CsA-C_0_ [μg/L]
221 ± 60	55 ± 87	54 ± 25
(n = 35)	(n = 35)	(n = 25)
	EVR dose [mg/m^2^]	EVR dose [mg/m^2^]
	1.4 ± 0.6	1.3 ± 0.6
	EVR-C_0_ [μg/L]	EVR-C_0_ [μg/L]
	4.2 ± 1.3	4.6 ± 1.9
	(n = 35)	(n = 35)
**Control group**		
TAC dose [mg/m^2^]	TAC dose [mg/m^2^]	TAC dose [mg/m^2^]
9.2 ± 2.9	4.4 ± 2.6	3.8 ± 2.0
TAC-C_0_ [μg/L]	TAC-C_0_ [μg/L]	TAC-C_0_ [μg/L]
9.2 ± 4.4	6.4 ± 2.0	6.7 ± 5.5
(n = 56)	(n = 64)	(n = 64)
MMF dose [mg/m^2^]	MMF dose [mg/m^2^]	MMF dose [mg/m^2^]
756 ± 492	579 ± 217	521 ± 184
n = 70)	(n = 54)	(n = 53)

C_0_, trough level; CsA, cyclosporine; EVR, everolimus; MMF, mycophenolate mofetil; TAC, tacrolimus

The control group received an immunosuppressive regimen which represents the standard of care for pediatric renal transplant recipients in Europe, i.e. a CNI-based regimen (CsA or TAC) in conjunction with MMF and steroids. Only 4/70 patients (5.7%) received basiliximab. In the other 66 patients no induction therapy was administered. All children received 300 mg methylprednisolone/m^2^ BSA intravenously at the time of engrafting. The details of prednisolone dosing during the study period are described in the results section. Fifty-six children received TAC at an initial dose of 0.3 mg/kg body weight (b.w.) per day given in two divided doses, 14 children received CsA at an initial dose of 400 mg/m^2^ BSA per day given in two divided doses. Subsequent doses were adjusted based on the following target trough level ranges: TAC, 10–12 μg/L (days 0–21), 8–10 μg/L (days 22–183) and 5–10 μg/L (day 183 onwards); CsA, 140–200 μg/L (days 0–183) and 70–140 μg/L (from day 183 onwards). The target trough level ranges were modified based on clinical evidence of efficacy and occurrence of adverse events. The respective doses and trough levels of TAC and CsA are given in Table 1. In conjunction with TAC, the daily MMF dose was 1200 mg/m^2^ BSA for the first two weeks post-transplant, administered in two daily doses. Thereafter, the daily dose was 600 mg/m2 BSA given in two doses and adjusted if medically indicated. In conjunction with CsA, the MMF dose of 1200 mg/m² BSA per day was maintained. The respective immunosuppressant mean doses and corresponding trough levels are given in Table 1.

### Database

Data of both the EVR and the control group were registered retrospectively from patient files and entered into the CERTAIN Registry (www.certain-registry.eu) [9]. Because of its detailed and comprehensive data capture, CERTAIN allows an in-depth characterization of specific patient cohorts. Follow-up data were collected prior to engraftment, at month 1, 6 and 12 post-transplant and every 12 months thereafter. The detailed analysis plan is given in S2 Table. The data sets of the control group and the EVR group are available from the author LP. All patients and/or their parents/guardians provided written informed consent to participate in the registry. The CERTAIN Registry is kept in full accordance with the principles of the Declaration of Helsinki and Good Clinical Practice guidelines and approved by the ethics committee of each contributing center. The ethics committee of Hannover Medical School and the ethics committee of the University of Heidelberg approved this particular study.

### Outcome variables

The estimated glomerular filtration rate (eGFR) was calculated by the updated abbreviated Schwartz formula [10]. Serum creatinine was measured by an Integrated Database Management System traceable enzymatic assay (Roche Diagnostics, Mannheim, Germany). The Schwartz formula was also used for patients aged ≥18 years to adhere to the same eGFR formula throughout the observation period. Replication of cytomegalovirus (CMV), Epstein-Barr virus (EBV), and BK polyomavirus (BKV) was monitored by blood polymerase chain reactions (PCR) every three months in the first year post-transplant and at least every 6 months thereafter.

The panel reactive antibody (PRA) value was defined as the percentage of panel cells that reacts with patient serum in the complement-dependent cytotoxicity screening. Human leukocyte antigen antibodies were measured prior to engraftment and at least annually post-transplant by the LABScreen single-antigen beads Luminex kit (One Lambda, Canoga Park, CA) which uses single HLA-coated beads and enables identification of IgG alloantibody specificities against HLA-A, -B, -C, -DRB1/3/4/5, -DQA1, -DQB1, -DPA1 and -DPB1 antigens. Because no clinically validated cut-off for the Luminex assay is recommended by the provider company, a mean fluorescence intensity of ≥ 1000 was used to define the cut-off for antibody positivity. For high-resolution typing CTS-Sequence kits (Heidelberg, Germany) and Olerup-SSP kits (Saltsjöbaden, Sweden) were used.

Acute rejection episodes (ARE) were categorized as follows: (i) Biopsy-proven acute rejection (BPAR) Banff score ≥ IA on indication biopsy [11]; (ii) BPAR including borderline findings on indication biopsy, which triggered anti-rejection therapy; (iii) over-all treated ARE (BPAR plus ARE, where a graft biopsy was either logistically not possible or medically contraindicated, but where anti-rejection therapy was initiated). An infection was defined as any infection leading to specific antimicrobial treatment and/or hospitalization, corresponding to the definition of infection in the CERTAIN Registry.

Systolic and diastolic blood pressures were derived from casual blood pressure measurements (average of three measurements taken within 5 min) by sphygmomanometry. The standard deviation scores (SDS) for systolic and diastolic blood pressure and longitudinal growth was calculated using the Lambda Mu and Sigma equation taken from Cole [12]. Growth data from the German KiGGS study for the health of children and adolescents in Germany (“Studie zur Gesundheit von Kindern und Jugendlichen in Deutschland”) for the period 2003–2006 were used as a reference [13].

### Statistical analysis

For comparison o f independent groups regarding continuous variables with approximate normal distribution, the two-sided t-test was used (with Satterthwaite approximation in case of heterogeneous variances); not-normally distributed variables were analyzed with the Wilcoxon test. For categorical variables the chi-square test or, in case of small group sizes, the Fisher’s exact test was used. Patient and graft survival were evaluated by Kaplan-Meier analysis. IBM SPSS statistics 21 (IBM, Hannover, Germany) was used for the statistical analyses. Because of the hypothesis-generating character of this study, the p-values are regarded as descriptive.

## Results

### Study population

Data on the primary renal disease in both groups are given in S1 Table and demographic data in Table 2. In addition to the five matching criteria, the EVR and control group were also comparable regarding donor age, cold ischemia time, number of HLA mismatches and CMV serostatus prior to transplantation. All living-related donors were haploidentical; there were no HLA-identical donors. PRA values prior to transplantation (EVR group; median 0%, range, 0–28%; control group, median 0%; range, 0–92%) were comparable between groups (p = 0.82, Wilcoxon test). PRA values >6% as a surrogate marker of immunological high risk were present prior to transplantation only in 2/35 patients (5.7%) in the EVR group (PRA values of 16% and 28%, respectively) and in 3/70 (4.3%) patients in the control group (PRA values of 28%, 42% and 92%, respectively; p = 1.00, Fisher’s exact test). Preformed DSA were not present in any patient prior to transplantation.

**Table 2:** Patient characteristics at baseline.

Parameter	EVR group		Control group		P
Mean age at RTx* (years)	10.7 ± 5.6		9.8 ± 5.4		0.37
Male gender^#^	63%		64%		0.89
Living-related/ deceased donor#	57% / 43%		42% / 58%		0.13
Preemptive RTx^#^	30%		20%		1.0
Median donor age° (years)	34 (range, 2–50)		40 (range, 1–64)		1.0
Median cold ischemia time° (hours)	8.5 (range, 1.7–26)		9.8 (range, 0.75–26)		0.6
Number of HLA mismatches§	0	9%	0	5%	
	1	23%	1	20%	
	2	26%	2	41%	
	3	34%	3	28%	
	4	5%	4	2%	
	5	3%	5	2%	
Number of HLA-DR mismatches§	0	34%	0	24%	
	1	66%	5	2%	
	2	0%	2	10%	0.84
CMV serostatus prior to RTx^§^	D-/R-	13/35 (37%)	D-/R-	29/70 (42%)	0.83
	D-/R+	1/35 (3%)	D-/R+	8/70 (11%)	0.14
	D+/R-	12/35 (34%)	D+/R-	17/70 (24%)	0.28
	D+/R+	9/35 (26%)	D+/R+	16/70 (23%)	0.10

CMV, cytomegalovirus; HLA, human leukocyte antigen; RTx, renal transplantation: D, Donor; R, Recipient

(p calculated by t-test, Wilcoxon-test, chi-square-test or Fisher’s exact test).

*two sample t-test,

^#^chi-square test,

^°^Wilcoxon test,

^§^Fisher’s exact test.

For two patients of the EVR group, complete follow-up data were not available after transfer to adult care. Therefore in the EVR group, the complete data set was available for 35/35 patients at month 12, 18, and 24, and for 33/35 patients at month 36 and 48 post-transplant. No adjustments were made for missing data. Data on patient and graft survival were available for all patients at all time points. In the matched control group the data set was complete.

### Immunosuppressive therapy

In the EVR group, prednisolone was discontinued in 29/35 (83%) patients at 10±2 months post-transplant. Prednisolone treatment was only maintained in patients with a history of acute rejection. The corticosteroid dose was slowly tapered over a 12 week period until cessation. In the control group, steroids were withdrawn in 27/70 children (39%) at 17±15 months post-transplant. Three patients (4%) underwent early steroid withdrawal on day 5 post-transplant. Because steroid withdrawal was protocol-driven in all patients the EVR group but only in 3 children in the control group, the percentage of patients undergoing steroid withdrawal in the EVR group was higher (83% vs. 39%, p<0.001, Fisher’s exact-test) and the point in time post-transplant was earlier (10±2 vs. 17±15 months post-transplant, p = 0.01, t-test) than in controls. In 5/35 patients (14%) in the EVR group, prednisolone was reintroduced during the 4 year study period because of BPAR (borderline rejection or acute T cell mediated rejection) (n = 4), or because of late-onset adrenogenital syndrome (n = 1). Prednisolone was reintroduced in three patients in the control group (n = 1 borderline rejection, n = 1 acute T-cell mediated rejection, n = 1 during pregnancy).

**Fig 1:**
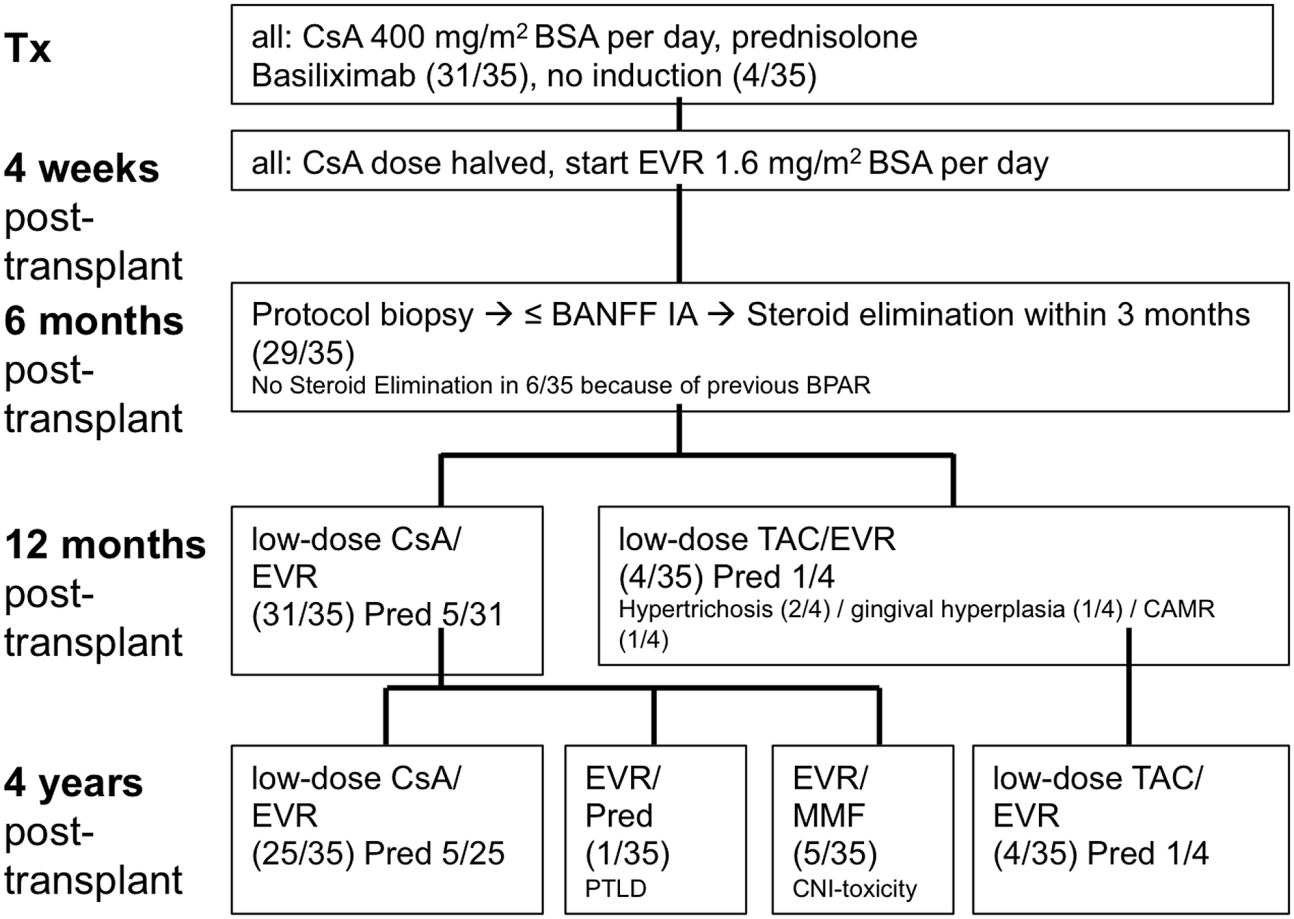
Course of immunosuppressive therapy in the EVR group (a). CsA, cyclosporine; DSA, Donor Specific Antibodies; EVR, everolimus; BSA, body surface area;* Tx, transplantation; Pred, Prednisolone; TAC, tacrolimus; MMF, mycophenolate mofetil, PTLD, post transplant lymphoproliferative disease; CAMR, chronic antibody mediated rejection.

In the EVR group, 31/35 patients (88%) received treatment with low-dose CsA and EVR at one year post-transplant. The immunosuppressive therapy was switched in four patients (11%) from CsA to TAC because of CsA-related side effects (gingival hyperplasia or hypertrichosis, n = 3) or because of chronic antibody-mediated rejection (n = 1). At year 3 and 4 post-transplant, 25/33 patients (76%) were treated with the initial immunosuppressive regimen, 4/33 patients (12%) received TAC and EVR and 5/33 patients (15%) received EVR and MMF because of biopsy-proven CNI-induced chronic nephrotoxicity. One patient of the EVR group developed post-transplant lymphoproliferative disease (PTLD); CsA was discontinued and EVR plus prednisolone was given. In 5/35 patients (14%), EVR treatment was temporarily discontinued and CsA dose was doubled for 2–4 weeks because of impaired wound healing (n = 2) or major surgery (n = 3). The course of immunosuppressive therapy in the EVR group is depicted in Fig 1.

In the control group, 56 patients (80%) received initially a regimen of TAC and MMF and 14 patients (20%) a regimen of CsA and MMF. Eight of these 14 patients (57%) were later switched to a TAC-based regimen due to BPAR including borderline rejection. In one patient TAC was switched to CsA at day 30 post-transplant because of TAC-induced left ventricular hypertrophy. In one patient immunosuppressive therapy was switched from TAC plus MMF to EVR monotherapy at month 9 post-transplant because of BK nephropathy, in two patients from CsA plus MMF to TAC plus MMF because of hypertrichosis at year 1 or detection of DSA at year 3 post-transplant. Because of CNI-induced chronic nephrotoxicity (n = 2), immunosuppressive therapy was changed in one patient from CsA plus MMF to low-dose CsA plus EVR at year 4 and in one patient from TAC plus MMF to low-dose TAC plus EVR at month 8 post-transplant. MMF was permanently discontinued in 13/70 patients (19%) because of side effects (gastrointestinal (n = 3), warts (n = 2), BKV-nephropathy (n = 1), BK-viremia (n = 1), leukopenia (n = 1), rhabdomyosarcoma (n = 1) or unspecified/unknown (n = 4)). In 4 of these 13 patients (31%) azathioprine was administered instead of MMF. Two patients in the control group developed PTLD; TAC was discontinued, and immunosuppressive therapy was carried out with MMF and methylprednisolone. In one of these PTLD patients, EVR was added to the immunosuppressive regimen 2.3 years after the diagnosis of PTLD. In one patient CsA was reintroduced 6 months after diagnosis of PTLD.

### Patient and graft survival

In the EVR group, patient and graft survival at year 4 post-transplant was 100%. One of two FSGS patients experienced a recurrence of this primary renal disease; the graft was salvaged by intense immunoadsorption therapy. In the control group, one child died in the fourth year post-transplant due to cerebral edema and multi-organ failure because of treatment-resistant septicemia, still with residual graft function.

### Rejections

Within the observation period of 4 years, 16/35 patients (46%) in the EVR group and 50/70 patients (71%) in the control group (p = 0.02, chi-square test) underwent a renal graft biopsy for clinical indication. Twenty-nine indication biopsies were performed in 16 patients of the EVR group (1.8 biopsies per patient) and 102 indication biopsies in 50 patients of the control group (2.0 biopsies per patient). Biopsy results in the first year post-transplant are given in Table 3. For all three analyzed ARE categories, the frequency was numerically higher in the control group. BPAR including borderline findings in indication biopsies, treated with anti-rejection therapy during the first year post-transplant, were observed in 6/35 (17%) patients in the EVR group and in 24/70 (34%) patients in the control group (p = 0.07, Chi square test). In both groups, no patient received anti-rejection therapy for presumed rejection. Only a few patients experienced ARE after the first year post-transplant: EVR group, 1/35 (3%); control group, 6/70 (8.6%) (p = 0.42, Fisher’s exact test). Chronic antibody-mediated rejection as defined by Banff 2007 criteria was detected in one patient in each treatment group.

**Table 3:** Results of indication biopsies during the first year post-transplant.

	EVR group	Control group	P value
	(n = 35)	(n = 70)	
N	16 (46%)	44 (63%)	0.10
BPAR (BANFF ≥ IA)	2 (6%)	8 (11%)	0.49
BPAR including treated borderline findings	6 (17%)	24 (24%)	0.07
Overall treated ARE	6 (17%)	24 (34%)	0.07
Acute tubular necrosis	4 (12%)	5 (7%)	0.48
Interstitial fibrosis	3 (9%)	4 (6%)	0.68
CNI-induced chronic nephrotoxicity	0 (0%)	3 /4%)	0.54
Recurrent MPGN	1 (3%)	0 (0%)	0.33

CNI, calcineurin inhibitor; MPGN, membranoproliferative glomerulonephritis.

p values calculated by Fischer’s exact test. The respective numbers refer to number of patients. Acute Rejection Episodes (ARE) were defined as follows: (i) Biopsy-proven acute rejection episodes (BPAR) BANFF score ≥ IA on indication biopsy (11); (ii) BPAR including borderline findings on indication biopsy, which triggered anti-rejection therapy; (iii) over-all treated ARE (BPAR plus ARE, where a graft biopsy was either logistically not possible or medically contraindicated).

### Donor-specific HLA antibodies

Four of 35 patients (11.4%) in the EVR group exhibited *de novo* donor-specific HLA antibodies (DSA) either at year 3 (n = 2) or year 4 (n = 2) post-transplant. In the control group, yearly DSA measurements were available in 56/70 children. Ten of 56 patients (17.9%) developed de novo DSA either at year 1 (n = 2), year 2 (n = 1), year 3 (n = 2) or year 4 (n = 5) post-transplant (p = 0.55, Fisher’s exact test).

### Kidney function

**Fig 2:**
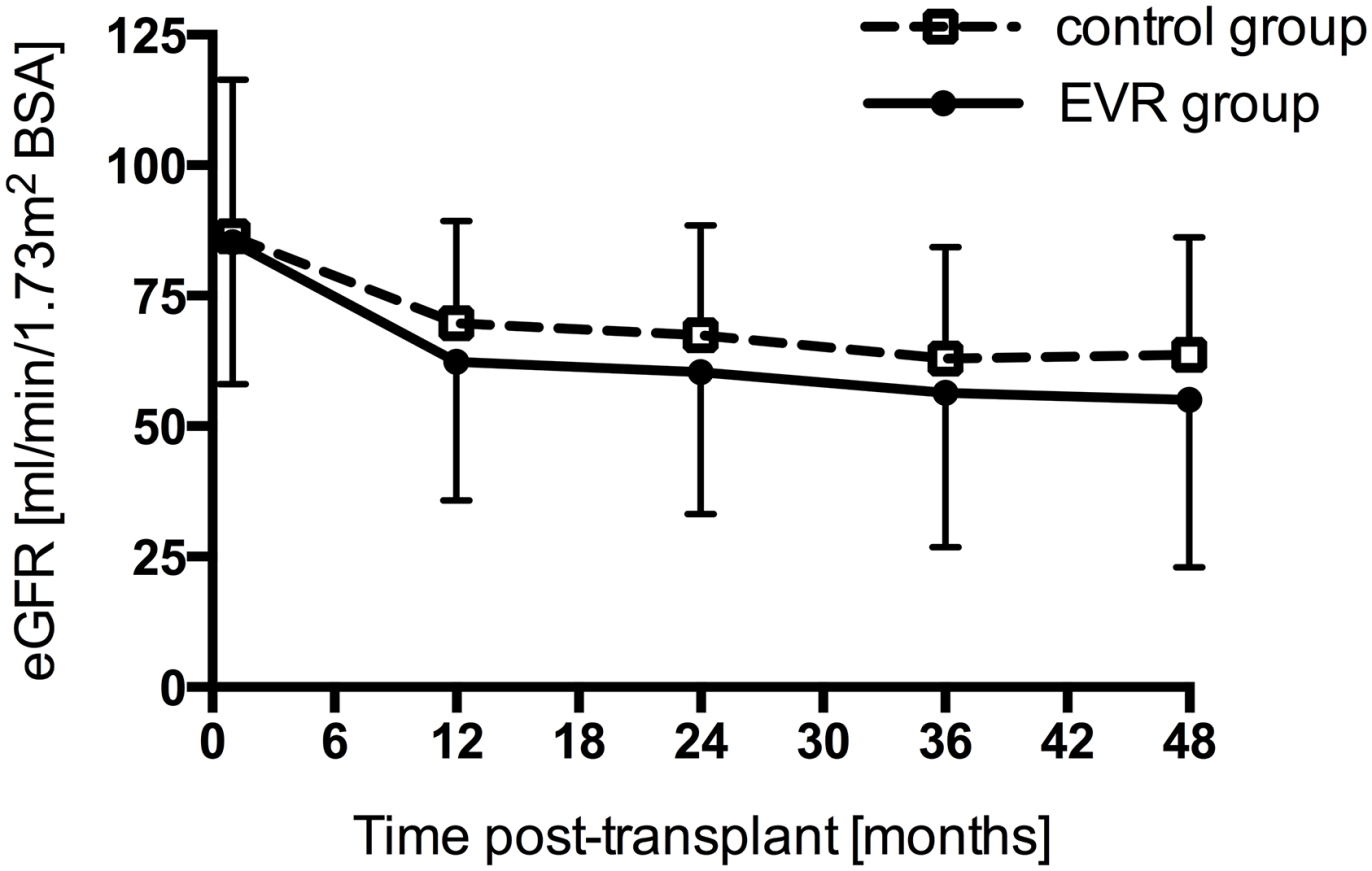
Estimated glomerular filtration rate (eGFR) over the observation period of 4 years in the everolimus (EVR) group (n = 35 until year 2; n = 33 at year 3 and 4) and the control group (n = 70). Data are mean ± SD. BSA, body surface area.

At baseline, defined as month 1 post-transplant, mean eGFR was similar between the groups (Fig 1). During the first year post-transplant, there was a numerically higher decline of eGFR in the EVR group (p = 0.14, two-sample t-test). After year 1 post-transplant, the decline of eGFR was comparable between the two groups (Fig 2).

### Other laboratory analyses

In the EVR group, plasma cholesterol (by 26%), LDL-cholesterol (by 28%) and triglyceride levels (by 45%) were considerably higher than in the control group during the entire observation period (Table 4). Two of 35 patients in the EVR group and no patient in the control group were treated with pravastatin because of a plasma LDL-cholesterol >160 mg/dL (p = 0.11, Fisher’s exact test).

**Table 4:** Plasma triglyceride, cholesterol and low-density lipoprotein (LDL) cholesterol values and the urinary albumin/creatinine ratio in the everolimus (EVR) group (n = 35 until month 24 and n = 33 at month 36 and 48) and in the control group (n = 70).

Time post-transplant (months)	1	12	24	36	48
**Triglycerides (mg/dL)**					
EVR group	256 ± 152	212 ± 84	203 ± 122	237 ± 166	241 ± 213
Control group	233 ± 125	129 ± 99	129 ± 87	125 ± 70	132 ± 48
P value	0.41	< 0.001	< 0.001	< 0.001	< 0.001
**Cholesterol (mg/dL)**					
EVR group	243 ± 59	223 ± 75	214 ± 60	214 ± 47	222 ± 56
Control group	211 ± 43	166 ± 38	167 ± 40	165 ± 36	165 ± 36
P value	0.005	< 0.001	< 0.001	< 0.001	< 0.001
**LDL cholesterol (mg/dL)**					
EVR group	152 ± 46	138 ± 46	131 ± 39	135 ± 41	124 ± 30
Control group	104 ± 30	86 ± 33	89 ± 37	87 ± 29	89 ± 35
P value	< 0.001	< 0.001	< 0.001	< 0.001	< 0.001
**Albuminuria (mg/mmol creatinine)**					
EVR group	12 ± 19	9 ± 14	19 ± 40	21 ± 40	8 ± 5
Control group	14 ± 40	9 ± 25	11 ± 14	20 ± 61	12 ± 31
P value	0.87	1.0	0.83	0.93	0.45

Month 1: Values determined before initiation of EVR therapy.

P values were calculated by the two-sample t-test.

The urinary albumin/creatinine ratio was low in both groups and comparable (Table 4). Beside one patient with FSGS recurrence, no patient experienced an urinary albumin/creatinine ratio above 100 mg/mmol. No patient was treated with an ACE-inhibitor due to proteinuria.

In the EVR group, the hemoglobin concentration was numerically lower than in the control group at most of the study time points (Table 5), p = 1.00, 0.09, 0.08, 0.01 and 0.31 for baseline and year 1, 2, 3 and 4). The EVR group also received more frequently treatment with erythropoiesis-stimulating agents. There were no differences in white blood cell counts between the groups (Table 5).

**Table 5:** Hemoglobin concentration, treatment with erythropoiesis-stimulating agents (ESA) and leukocyte counts in the everolimus (EVR) group (n = 35 until month 24, n = 33 at months 36 and 48) and the control group (n = 70).

Time post-transplant (months)	1	12	24	36	48
**Hemoglobin (mg/dL)**					
EVR group	11.5 ± 1.9	11.1 ± 1.0	10.7 ± 1.5	11.5 ± 0.9	12.2 ± 1.0
Control group	9.9 ± 1.1	11.8 ± 1.5	12.2 ± 1.6	12.2 ± 1.5	12.5 ± 1.6
P value	1.0	0.09	0.08	0.01	0.31
**Leukocytes (/μL)**					
EVR group	12.0 ± 4.2	7.9 ± 2.2	8.1 ± 3.0	8.5 ± 3.0	8.2 ± 3.6
Control group	12.2 ± 5.3	8.5 ± 3.9	8.8 ± 3.2	9.0 ± 2.9	8.9 ± 3.1
P value	0.85	0.40	0.28	0.42	0.30
**ESA treatment (%)**					
EVR group	3%	23%	34%	26%	29%
Control group	4%	10%	16%	14%	12%
P value	1.0	.025	0.04	0.17	0.06

Erythropoetin alpha, beta and zeta, Darbepoetin alpha and Methoxy-polyethylene-glycol-epoetin beta were summarized as erythropoiesis-stimulating agents (ESA). Month 1: values determined before initiation of EVR. P values were calculated by the two-sample t-test (hemoglobin, leukocyte count) and by the Fisher’s exact test for the ESA treatment.

### Infections and hospitalizations

The rates of infectious episodes and hospitalizations were comparable between the groups (Table 6). Although in the EVR group no CMV-prophylaxis was administered, only 1/12 patients (8%) at high risk for CMV had asymptomatic CMV-replication; no symptomatic CMV-infections were observed. In the control group, 3/17 patients (18%, p = 0.62, Fisher’s exact test) at high CMV-risk developed CMV-syndrome after cessation of 3 months chemoprophylaxis with valganciclovir. Primary EBV infection or reactivation was found in 10/35 (29%) patients in the EVR group and in 9/70 (13%) in the control group (p = 0.25, chi-square test). One patient in the EVR group (3%) exhibited a positive whole blood PCR for BKV at month 12 post-transplant and spontaneous remission after 4 weeks. There was no patient with biopsy proven BK nephropathy in the EVR group. In the control group, a positive whole blood PCR for BKV was found in 12/70 (17%) patients (p = 0.04, Fisher’s exact test) requiring reduction of immunosuppression. In the control group one case of BKV nephropathy was observed on graft biopsy.

**Table 6:** Infectious episodes and hospitalization rates in the everolimus (EVR) group (n = 35 until month 24, n = 33 at month 36 and 48) and the control group (n = 70).

**Cause of infectious episodes**	**Rate per year$**	**Bacteria** ^**§**^	**Viruses** ^**§**^	**Funghi** ^**§**^	**Unknown** ^**§**^	
EVR group	1 (0–3)	70%	27%	3%	0%	
Control group	1 (0–4)	54%	26%	1%	19%	
P value	0.82	0.14	0.82	1.0	0.004	
**Location of infections**	**Urinary tract§**	**Upper airway§**	**Lower airway§**	**GI§**		
EVR group	17%	45%	26%	12%		
Control group	24%	20%	26%	30%		
P value	0.03	0.08	1.0	0.05		
**Reason for hospitalizations**	**Rate per year$**	**Infections§**	**GI§**	**Surgery§**	**ARE§**	**other**
EVR group	0.6 (range 0–3)	52%	24%	6%	5%	13%
Control group	0.59 (range 0–3)	28%	24%	12%	15%	21%
P value	0.93	0.22	1.0	0.49	0.21	0.43

Data are given as median (range) or as percentage. P values were calculated by the ^$^Wilcoxon test or by the ^§^Fischer’s exact test. ARE, acute rejection episodes; EVR, everolimus; GI, Gastrointestinal

One patient in the EVR group and two patients in the control group developed PTLD that was successfully treated with rituximab. One patient in the control group developed a rhabdomyosarcoma that was successfully treated by surgery and radiotherapy.

### Growth

**Fig 3:**
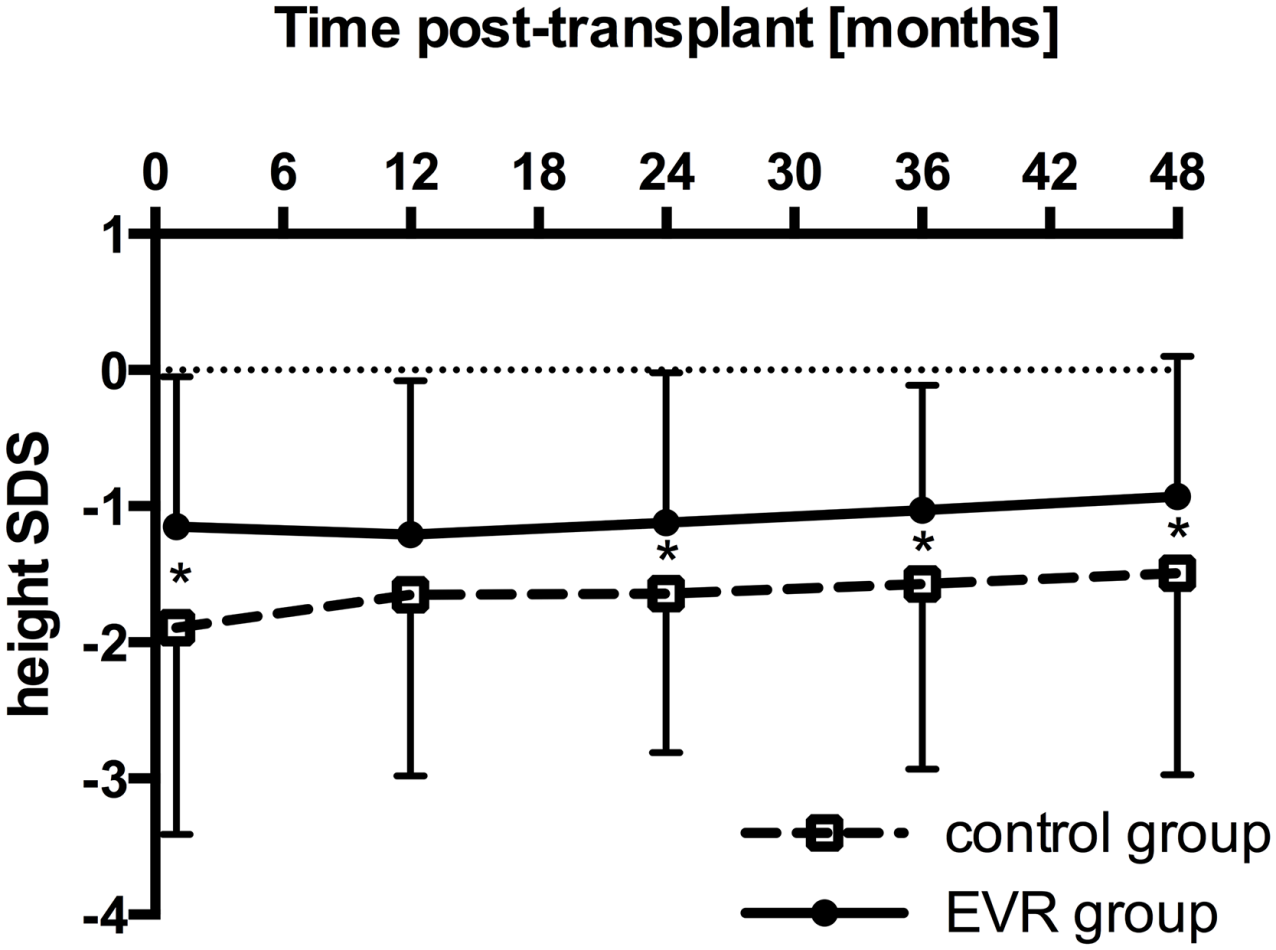
Height standard deviation score (SDS) over the observation period of 4 years in the everolimus (EVR) group (n = 35 until year 2; n = 33 at year 3 and 4) and the control group (n = 70). Data are mean ± SD. P values (by unpaired t-test) were as follows: p = 0.01 at baseline, p = 0.10 at year 1, p = 0.05 at year 2, p = 0.03 at year 3 and p = 0.05 at year 4 post-transplant. Significant differences (p ≤ 0.05) between the two groups are marked by an asterisk (*).

Growth during the observation period of 4 years was comparable between the groups (Fig 3). Recombinant human growth hormone was applied in no patient in the EVR group and in 4/70 patients (5.7%) in the control group and (p = 0.30, Fisher’s exact test).

### Sex hormones

In the EVR group, testosterone, estradiol, FSH and LH levels measured yearly were within the normal range according to chronological age in all male and female patients. In the control group, some transient changes in sex hormones were observed; at year 3 and 4 post-transplant, all sex hormone levels in the control group were within the normal range according to chronological age.

### Blood pressure

Mean standardized systolic and diastolic blood pressure were comparable in the two groups at most of the study time points (Table 7). The majority of patients (33/35, 94%) in the EVR group were treated with an ACE-inhibitor or angiotensin-receptor-antagonist from month 1 post-transplant on because of arterial hypertension, while in the control group only 49/70 patients (70%) were treated with any antihypertensive medication. Therefore, the percentage of antihypertensive patients, defined as either a blood pressure >2 SDS or antihypertensive medication administered, was clearly higher in the EVR group than in the control group during the first two years post-transplant.

**Table 7:** Height and blood pressure standard deviation scores (SDS) and antihypertensive treatment in the everolimus (EVR) group (n = 35 until month 24, n = 33 at month 36 and 48) and the control group (n = 70).

Time post-transplant (months)	1	12	24	36	48
**Height (SDS)**					
EVR group	-1.15 ± 1.10	-1.21 ± 1.13	-1.12 ± 1.10	-1.03 ± 0.92	-1.03 ± 0.92
Control group	-1.89 ± 1.52	-1.65 ± 1.33	-1.64 ± 1.37	-1.57 ± 1.36	-1.49 ± 1.48
P value	0.01	0.10	0.05	0.03	0.05
**Systolic blood pressure (SDS)**					
EVR group	1.61 ± 1.52	1.43 ± 1.68	1.14 ± 1.44	0.56 ± 1.55	1.20 ± 0.94
Control group	1.32 ± 1.55	0.88 ± 1.42	0.96 ± 1.23	0.96 ± 1.21	0.65 ± 1.24
P value	0.38	0.08	0.52	0.25	0.03
**Diastolic blood pressure (SDS)**					
EVR group	1.17 ± 1.10	1.05 ± 1.13	0.87 ± 1.10	0.16 ± 0.92	0.71 ± 1.03
Control group	0.77 ± 1.62	0.97 ± 1.48	0.53 ± 1.42	0.59 ± 1.35	0.54 ± 1.32
P value	0.25	0.86	0.31	0.28	0.59
**Relative percentage of hypertensive patients (%)** a					
EVR group	100%	100%	100%	85%	91%
Control group	83%	70%	72%	68%	69%
P value	< 0.01	< 0.01	0.001	0.126	0.049

^a^Arterial hypertension was defined as systolic and/or diastolic blood pressure > 95^th^ percentile and/or antihypertensive medication administered. P values were calculated by the t-test for unpaired samples.

### Surgical problems

No lymphoceles were detected in the EVR group and the control group. In the EVR group impaired wound healing was recognized in 2/35 patients; no impaired wound healing was reported in the control group.

## Discussion

This is the first long-term study on the efficacy and safety of an EVR-based immunosuppressive regimen in pediatric renal transplant recipients compared to a standard CNI- and MMF-based regimen. We observed that EVR, targeted to 3–6 ng/mL, in conjunction with low-dose CsA is associated with comparable efficacy outcome parameters such as graft and patient survival and renal graft function as the standard regimen. Attempts to minimize CNI exposure by reducing or eliminating CNIs from immunosuppressive regimens have previously been limited by an increase in acute rejection [14,15]. In the present study, the efficacy of the EVR-based regimen regarding the prevention of BPAR tended to be higher than the standard regimen. It is conceded that it is not possible to determine whether this is due to protection from basiliximab or the combination of EVR and CsA, as only a low number of patients was treated with basiliximab in the control group. However, the aim of our study was to compare the over-all efficacy of this new immunosuppressive regimen with the standard of care and not to dissect out the immunosuppressive potency of a single component (e.g. EVR) of this regimen. In addition, the rate of *de novo* DSA was comparable. Hence, CsA minimization facilitated by EVR co-administration appears to be a viable option in the treatment of *de novo* pediatric renal transplant recipients that maintains good efficacy. Despite CsA minimization and a lower rate of BPAR, renal function was not statistically different between the groups over the observation period of 4 years. However, it remains unclear, whether the numerically higher GFR in the control group might have any clinical relevance. Also in adult renal transplant recipients, *de novo* EVR treatment plus reduced dose CsA was not associated with a higher renal function at one year post-transplant compared to a standard-exposure CsA- and MPA-treated group [1,16]. Hence, the potential of an EVR-based regimen plus reduced-dose CNI for better long-term renal function may be somewhat limited.

The overall safety profiles were different between the groups. EVR therapy was associated with less BK polyomavirus replication, but with a higher percentage of arterial hypertension and more hyperlipidemia. Also the incidence of CMV replication in CMV high-risk patients in the EVR group was low, although no chemoprophylaxis with valganciclovir was administered. The association of an EVR-based regimen with a low rate of BKV and CMV replication has also been reported in studies in adult renal transplant recipients [17–21]. While hyperlipidemia is a known side effect of EVR [22], the higher percentage of hypertensive patients in the EVR group is more difficult to explain and may be a center effect. Some of the differences in hemoglobin concentrations between the groups may be due to the high rate (94%) of treatment with an ACE-inhibitor or angiotensin receptor antagonist in the EVR group. It is reassuring that low-dose EVR therapy, targeted to 3–6 ng/mL, was not associated with a higher degree of albuminuria than the standard regimen. Also longitudinal growth was comparable between the groups; but these data are difficult to interpret, because the percentage of steroid-free and growth-hormone treated patients was different between the two groups. We previously reported that longitudinal growth over 2 years in steroid-free pediatric patients on low-dose EVR and CsA is not different to that of a matched steroid-free control group on an immunosuppressive regimen with standard-dose CNI and MMF [23]. Hence, low-dose EVR does not appear to negatively impact longitudinal growth in pediatric renal transplant recipients.

The over-all tolerability of EVR in this study was good, with all patients still being on study drug at 4 years post-transplant. A previous single-arm, prospective multicenter trial on the efficacy and safety of combined low-dose CsA and EVR plus basiliximab induction and low-dose prednisone (alternate days from the sixth post-transplantation month) over 3 years reported a discontinuation rate of EVR of 32% [5]. In adult renal transplant recipients, a previous study on EVR, targeted to 3–8 ng/mL, in conjunction with reduced-dose CsA reported a study drug discontinuation rate of 23.4% compared to 15.8% in the control group [1]. The low discontinuation rate of EVR in our study may be due to the single-center use of EVR, the low risk patient profile with a high percentage of living-related donors (57%) and the low target blood level range for EVR (3–6 ng/mL).

We acknowledge some limitations of this observational study. Relatively small patient numbers, the lack of randomization and the exploratory study design make it difficult to draw definite conclusions. Steroid regimens and the percentage of patients treated with basiliximab induction therapy were not comparable between the groups. However, as noted above, the aim of our study was to compare the over-all efficacy and tolerability of this new immunosuppressive regimen with the standard of care and not to dissect out the impact of a single component (e.g. EVR) of this regimen. Since the study population was at low immunological-risk and a majority of patients were Caucasian with low PRA levels, these results may not be directly transferable to other transplant populations. It should be mentioned that a 36-month randomized marketing authorization study in *de novo* kidney transplant recipients, the CRADLE study (EudraCT 2010-024381-21) is currently ongoing [24]. This trial will evaluate whether early introduction of EVR with reduced TAC and steroid withdrawal offers similar efficacy and safety to a standard regimen of TAC, MMF and steroids, and whether renal function differs between the two groups.

In conclusion, this long-term multicenter, observational, matched cohort study in pediatric renal transplant recipients showed that EVR, targeted to 3–6 ng/mL plus reduced-dose CsA was as effective as a standard-dose CNI- and MMF-based regimen regarding patient and graft survival, prevention of BPAR and renal graft function. CsA minimization facilitated by EVR is therefore a viable option in the treatment of de novo pediatric renal transplant recipients that maintains efficacy and safety and has the potential to facilitate steroid withdrawal and reduce the risk of CMV and BK replication, which may be associated with long-term improvements in outcome. Long-term morbidity and mortality of pediatric patients might, however, be influenced by the higher frequency of arterial hypertension and dyslipidemia under EVR therapy.

## Supporting Information

S1 Table Analysis plan. (DOCX)

S2 Table Primary renal disease. (DOCX)
