# Supplementary material for: Efficacy and Safety of an Everolimus- vs. a Mycophenolate Mofetil-Based Regimen in Pediatric Renal Transplant Recipients
Source: PLoS One. 2015 Sep 25;10(9):e0135439. doi: 10.1371/journal.pone.0135439 (PMC4583261; doi:10.1371/journal.pone.0135439)
Supplement: S1 Table — (DOCX) [file pone.0135439.s001.docx]

**Supplementary table 1 – Analysis plan**

**Long-term Efficacy and Safety of an Everolimus- vs. a Mycophenolate Mofetil-based Regimen in Pediatric Renal Transplant Recipients: a Case-Control Study within the CERTAIN Registry**

Lena Brunkhorst, Alexander Fichtner, Britta Hoecker, Greta Burmeister, Thurid Ahlenstiel-Grunow, Kai Krupka, Martin Bald, Antonia Zapf, Burkhard Tönshoff, Lars Pape

1. **Study Aims**
   1. To determine the long-term (4 years post-transplant) efficacy and tolerability of a new immunosuppressive regimen consisting of basiliximab, low-dose cyclosporine A, everolimus and steroid elimination within the first year post-transplant compared to a standard regimen consisting of a calcineurin inhibitor, mycophenolate mofetil and maintenance steroids. Patients with steroid withdrawal earlier than 6 months after transplantation were excluded.
2. **Study Cohort**
   1. The Everolimus Cohort consists of 35 patients who have received an immunosuppressive regimen as described in 1.1. For each patient in the everolimus group, two case-control counterparts with a standard immunosuppressive regimen as described in 1.1 are identified within the CERTAIN Registry (Cooperative European Paediatric Renal Transplant Initiative) by means of the following five matching criteria: (i) age at renal transplantation, (ii) graft source (living donation or donation from a deceased donor), (iii) first or second transplant, (iv) gender, (v) pre-emptive transplantation or dialysis prior to transplantation. In both groups data requirements are the following: a complete CERTAIN minimal data set for at least the first four years post-transplant (initial visit prior to transplantation, day of discharge (30 days post-transplant) visit, 12, 24, 36 and 48 months post-transplant).
3. **Parameters of specific interest**
   1. **Patient and transplant characteristics**
      1. Demographic parameters (age, gender, height, weight, primary kidney disease).
      2. Transplant data (number of transplants, living or deceased donation, cold ischemia time, number of HLA mismatches, date of transplantation).
      3. Immunosuppressive medication (type of immunosuppressants, dosage, trough levels, periods of administration).
      4. Cytomegalovirus (CMV) risk group (CMV serology of donor (D) and recipient (R) prior to transplantation: CMV D+/R+, D+/R+, D-/R+, D-/R-).
      5. Transplant function: serum creatinine, height (eGFR (estimated glomerular filtration rate) according to Schwartz (JASN 2009) will be calculated).
      6. Treated and biopsy-proven acute rejection episodes (number, type according to the BANFF classification (Banff 97, 09 update)), date of onset, kind of treatment, outcome, steroid resistance; acute rejection episodes (ARE) are defined as follows: (i) Biopsy-proven acute rejection episodes (BPAR) BANFF score ≥ IA on indication biopsy (11); (ii) BPAR including borderline findings on indication biopsy, which triggered anti-rejection therapy; (iii) over-all treated ARE (BPAR plus ARE, where a graft biopsy was either logistically not possible or medically contraindicated, but where anti-rejection therapy was initiated). In addition, any biopsy-proven graft pathology according to the BANFF classification.
      7. Patient and graft survival
   2. **Infectious diseases**
      1. Viral diseases
         1. Any episode of CMV, (Epstein-Barr virus (EBV) and BK-polyomavirus (BKV) replication during the observation time. Documentation of treatment / outcome.
         2. Antiviral prophylaxis and therapy (agent, dose, duration).
      2. Other infectious diseases: any infection leading to treatment or hospitalization.
   3. **Hospitalizations**

Documentation of hospitalization episodes (cause and duration) per patient.

- 1. **Laboratory and anthropometric values**

Extraction of the following lab values out of the CERTAIN Registry at the time points given in 2.1.: urinary albumine, urinary creatinine, serum cholesterol, serum low-density lipoprotein cholesterol (LDL-C), serum triglycerides, complete blood count, trough levels of immunosuppressants, serum creatinine, height, weight.

- 1. **Medication**

Export of all medications administered during the observation time with start and end date from the CERTAIN Registry including immunosuppressants (with doses), antihypertensive drugs and erythropoiesis-stimulating agents**.**

- 1. **Blood Pressure**

Systolic and diastolic blood pressures were derived from casual blood pressure measurements (average of three measurements taken within 5 min) by sphygmomanometer and converted to SDS values based on reference data from the 2013 German healthy population (13).

- 1. **Donor-specific antibodies**

Documentation of the appearance of de novo donor-specific antibodies at the time points given in 2.1 determined by the LABScreen single-antigen beads Luminex kit. Because no clinically validated cut-off for the Luminex assay is recommended by the provider company, a mean fluorescence intensity of ≥ 1000 was used to define the cut-off for antibody positivity.
