# Supplementary material for: Efficacy and Safety of an Everolimus- vs. a Mycophenolate Mofetil-Based Regimen in Pediatric Renal Transplant Recipients
Source: PLoS One. 2015 Sep 25;10(9):e0135439. doi: 10.1371/journal.pone.0135439 (PMC4583261; doi:10.1371/journal.pone.0135439)
Supplement: S2 Table — (DOCX) [file pone.0135439.s002.docx]

**Supplementary Table 2: Primary renal disease**

|  |
| --- |

**EVR group Control p value**

**group**

|  |
| --- |

**n=35 n=70**

Renal hypo-/dysplasia^§^ n=11 n=21 1.00

Obstructive uropathy^§^ n=6 n=6 0.21

Nephronophthisis^§^ n=5 n=7 0.53

Autosomal recessive polycystic kidney disease^§^ n=3 n=6 0.95

Rapidly progressive glomerulonephritis^§^ n=3 n=4 0.58

Congenital nephrotic syndrome^§^ n=2 n=7 0.46

FSGS^§^ n=1 n=2 1.00

Membranoproliferative glomerulonephritis type 2^§^ n=1 n=0 0.16

Infantile nephropathic cystinosis^§^ n=1 n=1 0.61

Hemolytic uremic syndrome^§^ n=1 n=3 0.78

CKD of unknown cause^§^ n=0 n=6 0.07

Reflux nephropathy^§^ n=0 n=2 0.31

Other^§^ n=1 n=5 0.40

|  |
| --- |

CKD, chronic kidney disease; FSGS, focal-segmental glomerulosclerosis

P values were calculated by # chi-square test or § Fisher’s exact test.
